# The Circadian Clock Gene *Period1* Connects the Molecular Clock to Neural Activity in the Suprachiasmatic Nucleus

**DOI:** 10.1177/1759091415610761

**Published:** 2015-10-30

**Authors:** Takashi Kudo, Gene D. Block, Christopher S. Colwell

**Affiliations:** 1 Department of Psychiatry and Biobehavioral Sciences, University of California, Los Angeles, CA, USA; Takashi Kudo is now at Okinawa Institute of Science and Technology Graduate University, 1919-1 Tancha, Onna-son, Kunigami-gun, Okinawa 904-0495, Japan.

**Keywords:** BK currents, calcium, circadian, *Per1*

## Abstract

The neural activity patterns of suprachiasmatic nucleus (SCN) neurons are dynamically regulated throughout the circadian cycle with highest levels of spontaneous action potentials during the day. These rhythms in electrical activity are critical for the function of the circadian timing system and yet the mechanisms by which the molecular clockwork drives changes in the membrane are not well understood. In this study, we sought to examine how the clock gene *Period1* (*Per1*) regulates the electrical activity in the mouse SCN by transiently and selectively decreasing levels of PER1 through use of an antisense oligodeoxynucleotide. We found that this treatment effectively reduced SCN neural activity. Direct current injection to restore the normal membrane potential partially, but not completely, returned firing rate to normal levels. The antisense treatment also reduced baseline [Ca^2+^]i levels as measured by Fura2 imaging technique. Whole cell patch clamp recording techniques were used to examine which specific potassium currents were altered by the treatment. These recordings revealed that the large conductance [Ca^2+^]i-activated potassium currents were reduced in antisense-treated neurons and that blocking this current mimicked the effects of the anti-sense on SCN firing rate. These results indicate that the circadian clock gene *Per1* alters firing rate in SCN neurons and raise the possibility that the large conductance [Ca^2+^]i-activated channel is one of the targets.

## Introduction

Robust circadian rhythms are important to our health and wellness. Mechanistically, many cells within our body express circadian rhythms in cellular functioning that are generated by autoregulatory loops involving transcription, translation, and degradation of key *clock genes*. These rhythms have an endogenous periodicity of approximately 24 h (Hogenesch and Ueda, 2011; Brown et al., 2012; Buhr and Takahashi, 2013). Moreover, our bodies comprise an extensive network of circadian oscillators, with each of the major organ systems (e.g., heart, liver, pancreas) apparently having its own clockwork to regulate the transcription of genes that are important to the specific target organ (Dibner et al., 2010). These circadian rhythms are largely synchronized by central pacemaker neurons located in a small subset of cells in the hypothalamus of the central nervous system—known as the suprachiasmatic nucleus (SCN). These pacemaker neurons exhibit circadian rhythms in spontaneous firing rates (SFRs) which, as evidence indicates, are critical as an output for the circadian system (Colwell, 2011).

Previous studies implicate the molecular clockwork of SCN neurons in driving circadian rhythms in SCN electrical activity. For example, the *Tau* (Casein kinase 1ɛ) mutation in hamsters shortens the period of wheel-running activity and neural activity rhythms (Liu et al., 1997). Similarly, homozygote *Clock* mutant mice are behaviorally arrhythmic, while heterozygote animals show lengthened behavioral rhythms, findings that are paralleled by physiological recordings from the SCN (Herzog et al., 1998; Nakamura et al., 2002). Furthermore, *Cry1/2* double mutants show behavioral arrhythmicity and loss of rhythms in SCN neural activity (Albus et al., 2002). These correlative findings suggest that clock gene rhythms are translated into daily patterns of action potential activity in the SCN. However, the mechanisms by which the molecular clock regulates SCN neural activity are not known and are the focus of our present work.

In this study, we utilized antisense oligodeoxynucleotide (ODN) against *Period1* (*Per1*) as a tool to transiently suppress PER1 protein. We first confirmed that the treatment was selective and established a time course for the transient reduction in protein. Next, we determined the impact of the treatment on SFR and resting [Ca^2+^]i levels in SCN neurons. Finally, we measured the impact of the antisense treatment on three potassium currents known to modulate neural activity in the SCN including the fast delayed rectifier (FDR), A-type (I_A_), and large conductance [Ca^2+^]i-activated (BK) currents. The results indicate that circadian clock gene *Per1* rapidly alters the neural activity of SCN neurons and suggests that the BK current may be responsible.

## Material and Methods

### Ethical Approval

The experimental protocols used in this study were approved by the University of California, Los Angeles (UCLA) Animal Research Committee, and all recommendations for animal use and welfare, as dictated in the UCLA Division of laboratory Animals and the guidelines from the National Institutes of Health, were followed.

### Animals

We obtained C57BL/6 mice from breeding colonies at UCLA. For bioluminescence experiments, C57Bl/6 PER2::LUC knock-in mice (backcrossed for a minimum of 12 generations; Yoo et al., 2004) were used. Starting at 3 weeks of age, the mice were placed in chambers where lights came on at 9:00 a.m. and off at 9:00 p.m. (temperature, 22 ± 2℃). This LD cycle was used throughout the study with zeitgeber time (ZT) 0 defined as the time that the lights came on. Food and water were supplied ad libitum. Totally, 387 mice were used in this study.

### Brain Slice Preparation

Methods utilized were similar to those described previously (Kudo et al., 2011, 2013, 2014). Brain slices were prepared with standard techniques from mice (C57BL/6, PER2::LUC) at 1 month of age with mice killed at ZT 0. Mice were anesthetized by Isoflurane (Phoenix Pharmaceutical, Burlingame, CA) anesthesia and rapidly decapitated. To prepare SCN cultures, the brain was excised from skull and placed in chilled low Ca^2+^ ACSF [in mM: 26 NaHCO_3_, 1.25 NaH_2_PO_4_, 10 glucose, 125 NaCl, 3 KCl, 5 MgCl_2_, and 1 CaCl_2_, pH 7.2–7.4 (290–310 mOsm)]. After chilling, the brains were trimmed to a block containing the hypothalamus and optic nerves. The brain was sliced in coronal plane on a vibratome (Leica Microsystems, Buffalo Grove, IL) at a thickness of 200 µm. The slice was then trimmed to ∼4 × 4-mm squares, transferred directly to culture membranes (Merck Millipore, Darmstadt, Germany) in 35-mm culture dishes with 1 ml of MEM (Invitrogen, Carlsbad, CA) containing 30 mM HEPES, 20 mM D-glucose, 5% B27, 5 mM L-glutamine, and 25 U ml^−1^ streptomycin–penicillin, and maintained at 34℃ and 5% CO_2_ (Han et al., 2006).

### Immunohistochemistry

Mouse SCN slices were made with the same method described earlier (see Supplemental Figure 1 for immunohistochemistry [IHC] protocol). The SCN was fixed at ZT 2, 3, 4, 5, and 6, and PER1 expression was examined. The slices were fixed by 4% paraformaldehyde (Sigma-Aldrich) in phosphate-buffered saline (PBS; pH 7.4) at ZT 2, 3, 4, 5, and 6. After that the slices were fixed at 4℃ overnight and cryoprotected in 20% sucrose in PBS. IHC was performed on free-floating 20 µm cryostat (Thermo Fisher Scientific, Carlsbad, CA) coronal brain sections. Sections were then washed for 5 min with PBS (3 times) and dipped in 3% normal goat serum in PBS with 0.1% Triton X-100 for 1 h. Sections were incubated with PER1 (1:500; Thermo Fisher Scientific) or PER2 (1:400; Merck Millipore) antibody for 72 h at 4℃. PER2 antibody was established by LeSauter et al. (2012). Sections were rinsed 3 times (5 min) in PBS and incubated for 2 h with an Alexa Fluor 488 goat anti-rabbit IgG (Thermo Fisher Scientific) diluted 1:200 with Triton X-100-PBS with 3% normal goat serum. After incubation, sections were rinsed in TBS 3 times (5 min) and immediately mounted on slides. Sections were then dried and coverslipped. Then sections were imaged on the Axio Vision camera systems (Apotome, Carl Zeiss Microscopy, Thornwood, NY). Four sections from each slice were chosen and images were taken. Immune-positive cells within the SCN were counted manually at ×40 with the aid of a grid. All immune-positive cells within the grid were counted equally without regard to the intensity of the staining. Counts were performed by two observers blind to the treatment protocol, and the results were averaged. Relative optical density in the SCN was measured using Image J (NIH, Bethesda, MD) software.

**Figure 1.**
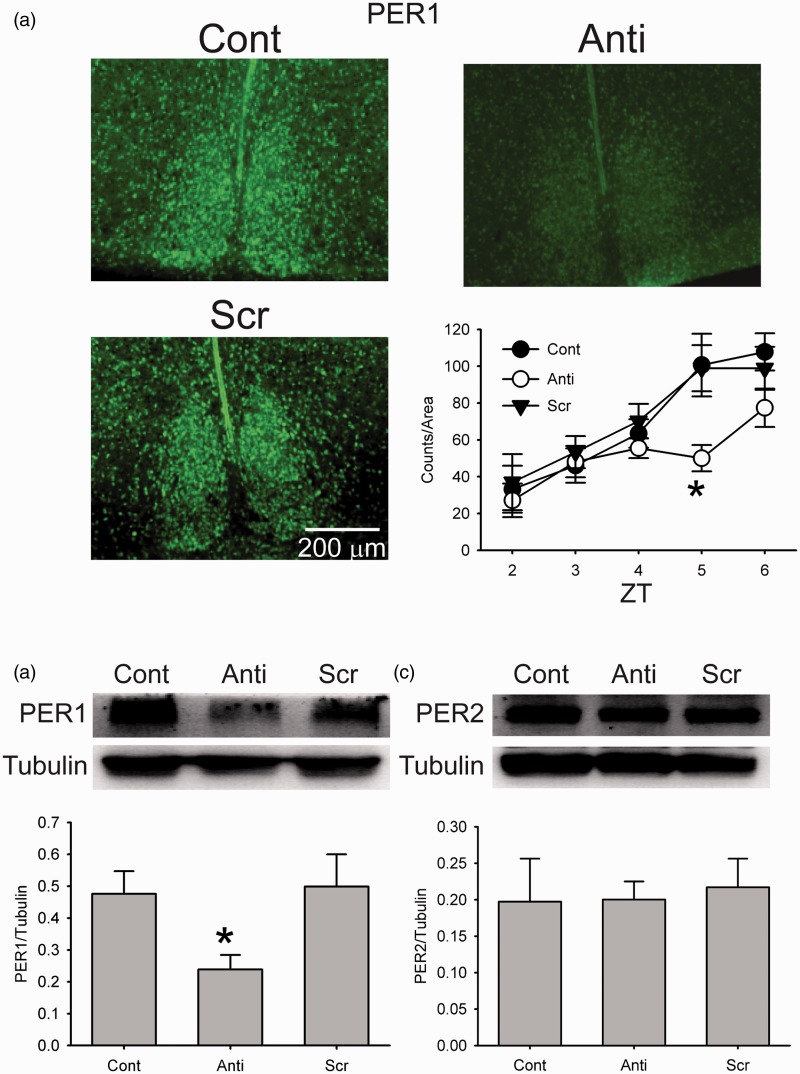
Antisense ODN for *Per1* reduced PER1 expression in the SCN. (a) Photomicrographs illustrate that the application of antisense ODN for *Per1* (10 µM) reduces PER1 expression as measured by immunohistochemistry (IHC). Cont: control, Anti: antisense ODN for *Per1*, and Scr: scrambled ODN. Bottom right, bar graphs show PER1 positive cell counts for Cont, Anti, and Scr (±*SE*, *n* = 3 for each time point). *Significant difference (*p* < .05) analyzed by two-way ANOVA followed by Holm-Sidak method for multiple comparisons (vs. Cont). ZT = Zeitgeber time. (b) Example of Western blots measuring PER1 expression in the SCN tissue. Tubulin protein expression was measured as a control for loading. Bottom: Levels of PER1 protein expression (+*SE*, normalized to tubulin, *n* = 3 for each group). *Significant difference (*p* < .05) analyzed by one-way ANOVA followed by Holm-Sidak method for multiple comparisons (vs. Cont). (c) Example of Western blots measuring PER2 expression in the SCN tissue. Tubulin protein expression was measured as a control for loading. Bottom: Levels of PER2 protein expression (+*SE*, normalized to tubulin, *n* = 3 for each group).

### Western Blotting

Methods used were similar to those described previously (Wang et al., 2009). Mouse SCN slices were made with the same method described earlier (see Supplemental Figure 1 for Western blot protocol). SCN were flash frozen in 50 µl of ice-cold homogenization buffer with protease inhibitors (Protease Inhibitors complete; Roche Molecular Biochemicals) at ZT 5. Protein concentration of the homogenized tissue was determined using the Bio-Rad Protein assay kit (Bio-Rad, Hercules, CA). Protein (8 µg) was electrophoresed on SDS/PAGE (15% gels), transferred on to nitrocellulose membranes, and probed with primary antibody (PER1 1:500, PER2 1:400, or tubulin 1: 2500, Merck KGaA) overnight. PER1 antibody is polyclonal antibody (catalog # PA1-524), and the original antibody epitope is PSLADDTDANSN. This antibody was used for Western blotting in several studies (Chilov et al., 2001; Gillespie et al., 2003; Muhlbauer et al., 2004; Shirogane et al., 2005; Muhlbauer et al., 2009; Gumz et al., 2010). The membranes were incubated with horseradish peroxidase-conjugated anti-mouse or anti-rabbit secondary IgG (1:1000), and protein signals were visualized by chemiluminescence (Immun-Star HRP detection kit; Bio-Rad). Normalized values were then expressed as the ratio of PER1 to tubulin or PER2 to tubulin protein expression.

### Loose Patch Recording

Methods used were similar to those described previously (Kudo et al., 2013). At ZT 4, slices were placed in a recording chamber attached to the stage of a fixed-stage upright differential interference contrast (DIC) microscope (Olympus, Center Valley, PA) and washed out with perfusion system until ZT 5. When the recoding was performed before ZT 5, the SCN slices were moved to a recording chamber and SFR was recoded (see Supplemental Figure 1 for protocol shown as electrophysiology 1). Using the voltage-clamp recording technique in the loose-patch configuration, we measured the SFR in SCN neurons. Each of these cells was determined to be within the dorsal region of the SCN by direct visualization of the location of the cell with infrared DIC microscopy. Recordings (1 min) were made during the day (ZT 4–8, 2–6 h after the treatment, and 0–4 h after washout). The slices were superfused continuously (2 ml min^−1^) with ACSF [in mM; 26 NaHCO_3_, 1.25 NaH_2_PO_4_, 10 glucose, 125 NaCl, 3 KCl, 2 MgCl_2_, 2 CaCl_2_, pH 7.2–7.4 (290–310 mosmol kg^−1^)] aerated with 95% O_2_–5% CO_2_. The loose-patch recordings from the dorsal SCN (dSCN) were taken with recording electrodes. We focused on this region of the SCN as the rhythms in electrical activity are more robust in this region of the SCN (Schaap et al., 1999). Micropipettes (3–5 MΩ) were pulled from glass capillaries (WPI, Sarasota, FL) on multistage puller (P-97; Sutter Instruments, Novato, CA) and filled with the ACSF solution. Recordings were obtained with Axopatch 200B amplifier (Molecular Devices, Sunnyvale, CA) and monitored online with pClamp (version 10.3, Molecular Devices). To minimize changes in offset potentials with changing solutions, the ground path used a KCl agar bridge. Each of the cells was determined to be within the SCN by direct visualization of the location of the cell with infrared (IR) DIC microscopy. All recordings were performed at room temperature during ZT 4 to ZT 8. Recordings were sampled at 10 kHz and filtered at 1 kHz. After the 1-min recordings in gap-free mode, the number of spike was counted. For some experiments, the ACSF perfusion solution containing Gabazine (20 µM) to block GABA_A_-mediated currents was applied in the bath for 5 min using bath application, and changes in SFR were measured. In some cases, SFR was calculated by the determining the mean SFR for all of the neurons recorded from that animal.

### Whole Cell Patch Recording

Methods used were similar to those described previously (Kudo et al., 2014). Using the whole cell patch configuration, we measured electrical property in SCN neurons (see Suplemental Figure 1 for protocol shown as electrophysiology 2). Each of these cells was determined to be within the dorsal region of the SCN by direct visualization of the location of the cell with infrared DIC microscopy. Recordings were made during the day (ZT 5–7, 3–5 h after the treatment, and 1–3 h after washout). Slices were placed in a recording chamber (PH-1, Warner Instruments) attached to the stage of a fixed-stage upright IR DIC microscope (Olympus). The slices were superfused continuously (2 ml min^−1^) with ACSF aerated with 95% O_2_–5% CO_2_. The whole cell patch-clamp recordings from the dSCN were taken with recording electrodes. These micropipettes (typically 7–9 MΩ) were pulled from glass capillaries (WPI) on a multistage puller (Sutter Instruments) and filled with the standard solution. The standard solution contained (in mM) 112.5 K-gluconate, 1 EGTA, 10 HEPES, 5 Mg ATP, 1 GTP, 0.1 leupeptin, 10 phosphocreatine, 4 NaCl, 17.5 KCl, 0.5 CaCl_2_, and 1 MgCl_2_. We calculated the free [Ca^2+^] concentration (Maxchelator software, Stanford University) to be 142 nM in the internal pipette solution. The pH was adjusted to 7.20–7.30, and the osmolality was adjusted between 290 and 300 mOsm. Recordings were obtained with an Axopatch 200B amplifier (Molecular Devices) and monitored online with pClamp (Clampex version 10.3, Molecular Devices). Each of the cells was determined to be within the dorsal region of the SCN by direct visualization of the cell’s location with DIC microscopy. Cells were approached with slight positive pressure. The pipette was lowered to the vicinity of the membrane while maintaining positive pressure. After a high-resistance seal (2–10 GΩ) was formed by application of negative pressure, a second pulse of negative pressure was used to break the membrane.

The access resistance of these cells ranged from 15 to 50 MΩ in the whole cell voltage-clamp configuration, while the cell capacitance was typically between 6 and 18 pF. Data were not collected if access resistance was >50 MΩ or if the value changed significantly (>20%) during the course of the experiment. The junction potentials between the pipette and the extracellular solution were canceled by the voltage offset of the amplifier before a seal was established and were not further corrected. Series and input resistance were monitored repeatedly by checking the response to small pulses in a passive potential range. Series resistance was not compensated, and the maximal voltage error that was due to this resistance was calculated to be 6 mV. The standard extracellular solution used for all experiments was ACSF. Recordings were sampled at 10 kHz and filtered at 1 kHz. Resting membrane potential was measured by averaging 1 min recording under TTX application. For current injection experiments, SFRs were measured in current clamp mode for 1 min at first. Next, 1 min square depolarizing current (5 pA) was injected to examine the effects of depolarization on SFR. When the membrane resistance is 1 GΩ, this current injection causes a 5 mV depolarization. After current injection, SFRs were recorded without current injection. Totally, 352 cells were recorded from 325 mice.

### Measurement of Specific Ion Currents

Mouse SCN slices were made with the same method described earlier. Using the whole cell patch configuration, we measured electrical property in SCN neurons (see Supplemental Figure 1 for protocol shown as electrophysiology 2). Each of these cells was determined to be within the dorsal region of the SCN by direct visualization of the location of the cell with infrared DIC microscopy. Recordings were made during the day (ZT 5–7, 3–5 h after the treatment, and 1–3 h after washout). Drug treatments were performed by dissolving pharmacological agents in the ACSF used to bathe the slices during recording. Solution exchanges within the slice were achieved by a rapid gravity-feed delivery system. For FDR and I_A_ potassium currents, 1,2-bis(o-aminophenoxy)ethane-N,N,N′,N′-tetraacetic acid (BAPTA, 1 mM) was added to the internal solution to buffer [Ca^2+^]i and inhibit [Ca^2+^]i-dependent potassium currents (Itri et al., 2010; Kudo et al., 2013). For the BK currents, minimal EGTA (0.5 mM) [Ca^2+^]i buffering of the internal solution was used to preserve [Ca^2+^]i influences, because BK currents is substantially influenced by [Ca^2+^]i concentration and Ca^2+^ fluxes through voltage-gated Ca^2+^ channels (Pitts et al., 2006).

Methods utilized for FDR currents were similar to those described previously (Itri et al., 2010). The FDR K^+^ currents were isolated pharmacologically with a voltage-step protocol in the whole cell voltage-clamp configuration with the neurons initially held at −70 mV. The protocol consisted of a 100-ms prepulse at −90 mV (to elicit maximal conductance) followed by 250-ms steps at progressively depolarized potentials (−80 to 50 mV, 10-mV steps). Current traces were recorded with pClamp software (Molecular Devices) with the whole cell voltage clamp recording configuration and then analyzed with Clampfit (Molecular Devices). Leak subtraction was performed during acquisition with a p/4 protocol, which utilizes four subpulses with one fourth the test pulse amplitude and reversed polarity given from a holding potential of −70 mV. Current traces from treatment were subtracted from control to isolate FDR currents. 4-Aminopuyridine (4-AP, 0.5 mM) and tetraethylammonium (1 mM) were used to isolate FDR currents. Current measurements were performed in control solution and after 5 min drug treatment in each cell. The ACSF perfusion solution contained Gabazine (20 µM) to block GABA_A_-mediated currents, cadmium (100 µM) to block calcium channels, TTX (2 µM) to block fast voltage-activated sodium channels.

Methods utilized for I_A_ currents were similar to those described previously (Itri et al., 2010). To isolate I_A_ currents in SCN neurons, two voltage-step protocols were used in the whole cell patch-clamp configuration. The first protocol consisted of a 100-ms prepulse at −90 mV (to elicit maximal conductance) followed by a 250-ms step at progressively depolarized potentials (−80 to 50 mV, 5-mV steps). The second protocol consisted of a 100-ms prepulse at −50 mV (to remove the contribution of the I_A_ current) followed by a 250-ms step at progressively depolarized potentials (−80 to 50 mV, 5-mV steps). Current traces from the second protocol were subtracted from the first to isolate I_A_ currents. The ACSF perfusion solution contained Gabazine (20 µM), cadmium (100 µM), TTX (2 µM).

Methods utilized for BK currents were similar to those described previously (Pitts et al., 2006). SCN neurons were voltage clamped at −60 mV and steady sate I–V curves were obtained at least 4 min after first obtaining a whole cell recording by stepping the potential from −100 mV to 80 mV at 20 mV increments using 180 ms steps. The ACSF perfusion solution contained Gabazine (20 µM), cadmium (100 µM), TTX (2 µM), and 4-AP (10 mM) to block FDR and I_A_ currents. Iberiotoxin (IbTX, 100 nM), a specific BK channel blocker (Galvez et al., 1990; Leonard et al., 1992; Giangiacomo et al., 1993; Garcia et al., 1997), was then added. Additional I–V curves were obtained 5 min later to measure the effect of IbTX on membrane currents. After the recordings, the washout was well performed for the next recording. All recordings were performed at room temperature. The currents were normalized by capacitance.

### [Ca^2+^]I Imaging

Methods used were similar to those described previously (Colwell, 2000). At ZT 4, the medium change was performed to wash drugs (see Supplemental Figure 1 for protocol shown as Ca^2+^ imaging). To load the dye into cells, membrane-permeable Fura2 acetoxymethyl (final concentration 50 µM, Thermo Fisher Scientific) was added to the SCN in the 35 mm dish and incubated at 34℃ for 40 min at ZT 4 (5% CO_2_ incubator). After that the slices were placed in a perfusion chamber (Warner Instruments) and superfused (2 ml min^−1^) with ACSF (room temperature) for 20 min. Slices were constantly oxygenated with 95% O_2_–5% CO_2_. The measurements were made between ZT 5 and ZT 7.

A cooled CCD camera (CoolSNAP ES, 1392 × 1040 pixel format, Photometrics, Tucson, AZ) was added to the fixed-stage microscope (BX50, Olympus) to measure fluorescence. The fluorescence of Fura2 was excited alternatively at wavelengths of 357 nm and 380 nm by means of a high-speed wavelength-switching device (Lambda DG-4; Sutter Instruments). Image analysis software (MetaFluor, Thermo Fisher Scientific) allowed the selection of several *regions of interest* within the field from which measurements are taken. To minimize bleaching, the intensity of excitation light and sampling frequency was kept as low as possible. Measurements were normally made once every 2 sec.

Free [Ca^2+^] was calculated from the ratio (*R*) of fluorescence at 357 and 380 nm, using the following equation: [Ca^2+^] = Kd × Sf × (*R−R*_min_)/(*R*_max_−*R*) (Grynkiewicz et al., 1985). The value of Kd was assumed to be 135 nM, while values for *R*_min_ and *R*_max_ were all determined via *in vitro* calibration methods (Molecular Probes). Once the calibrations were made, the camera settings were then fixed and measurements were made with 380 nm and 357 nm excitation of the solutions.

### Real-Time Monitoring of Bioluminescence

Methods used were similar to those described previously (Nakamura et al., 2011). Mouse SCN slices were made with the same method described earlier. At ZT 4, the medium change was performed to wash drugs. Explants were transferred on to Millicell membranes (0.4 µm, PICMORG50, Merck KGaA) resting on 1.2 ml of recording media that contained freshly added 0.1 mM luciferin (sodium salt monohydrate, Biosynth Chemistry & Biology, Itasca, IL), and the 35 mm dishes were sealed using autoclaved high-vacuum grease (Dow Corning, Midland, Michigan). Tissue explants were inserted in the LumiCycle photomultiplier tube (Actimetrics, Wilmette, IL), and bioluminescence was monitored at 37℃. The bioluminescence signal was counted in 1 min bins for every 10 min for at least 5 days without changing media. The data were normalized by subtraction of the 24 h running average from the raw data and then smoothed with a 2 h running average (LumiCycle analysis, Actimetrics).

### Drugs

4-AP, TTX, cadmium chloride, and IbTX were purchased from Sigma-Aldrich (St. Louis, MO). Gabazine (SR 95531 hydrobromide) was purchased from Tocris (Bristol, UK). For *Per1* gene knock down, either antisense ODN sequence against the 5′ translational start site of *Per1* (taggggaccactcatgtct; Sigma-Aldrich) or a random ODN with equivalent GC content (ccgttagtactgagctgac) was applied at a final concentration of 10 µM from ZT 2 to ZT 4. These antisense ODN and random ODN were previously used (Akiyama et al., 1999; Wakamatsu et al., 2001; Tischkau et al., 2003; Marquez et al., 2004; Koyanagi et al., 2006; Gamble et al., 2007; Kudo et al., 2013). While siRNA can be a more efficient method, there are problems of transfection efficiency while the ODN method to reduce *Per1* has been established in the SCN slices (Gamble et al., 2007; Kudo et al., 2013). The antisense ODN is incorporated into cells by endocytosis. The antisense ODN was applied from ZT 2 to ZT 4, because the SCN slices was moved from 35-mm culture dish to the perfusion chamber. In the perfusion chamber, the flow rate is 2 ml min^−1^ and it is cost prohibitive to continually apply antisense ODN through the perfusion system.

### Statistical Analysis

The data sets were analyzed by test for equal variance and normal distribution to help select the appropriate test. The data sets were analyzed by one-way, two-way, or two-way repeated-measures ANOVA. If significant group differences were detected by the ANOVA, then a post hoc analysis was applied. Statistical significances between two groups were determined by Student’s *t*-test. If equal variance and normal distribution tests were failed, Mann-Whitney *U* test was performed. Equal variance test was performed to check variances of SFR. Values were considered significantly different if *p* < .05. All tests were performed with SigmaPlot (version 12, Systat Software, Chicago, IL). Values are shown as means ±*SE* or +*SE*.

## Results

### Antisense ODN Against *Per1* Reduced PER1 Expression

To confirm the effectiveness of the antisense ODN against *Per1*, we used IHC to measure PER1 expression in the SCN brain slice (protocol shown in Supplemental Figure 1). The SCN was fixed at ZT 2, 3, 4, 5, and 6, and PER1 expression was examined. We applied two-way ANOVA, and there were main effects of time, *F*(4, 30) = 22.321, *p* < .01, and treatment, *F*(2, 30) = 7.337, *p* < .01, but there were no interactions of time x treatment, *F*(8, 30) = 1.588, not significant (n.s.). The application of antisense ODN for *Per1* (10 µM) at ZT 2 significantly reduced the number of PER1 expressing cell in the SCN at ZT 5 (Figure 1(a), two-way ANOVA followed by Holm-Sidak method for multiple comparisons, vs. Cont, *p* < .05). At ZT 5, we found that the number of PER1 expressing neurons was reduced by approximately 50% (Cont: 101 ± 17 PER1 positive cells/slice, Anti: 50 ± 7). Optical density measurements revealed a similar level of decreased expression (Cont: 876 ± 67 arbitrary unit, Anti: 470 ± 73 arbitrary unit; unpaired Student’s *t*-test: *p* < .05). In contrast, the scrambled ODN did not alter PER1 expression (two-way ANOVA followed by Holm-Sidak method for multiple comparisons, n.s.). As a control for the specificity of the antisense ODN against *Per1*, we also examined PER2 expression at ZT 5. We found no change in this closely related protein in the SCN (Cont: 34 ± 7 PER2 positive cells/slice; Treated: 31 ± 3; unpaired Student’s *t*-test, n.s.). To further analyze the clock gene expression, Western blots were performed (protocol shown in Supplemental Figure 1). As for PER1, there was significant main effects (one-way ANOVA, *F*(2, 6) = 3.605, *p* < .05) and again, antisense ODN against *Per1* significantly reduced the PER1 expression in the SCN (Figure 1(b), one-way ANOVA followed by Holm-Sidak, *F*(2, 6) = 3.905, *p* < .05) without altering PER2 expression (Figure 1(c), one-way ANOVA, *F*(2, 6) = 0.0611, n.s.). These results confirmed that antisense ODN for *Per1* is specifically suppresses PER1 protein expression.

### Antisense ODN Against *Per1* Reduced SFR in dSCN Neurons During the Day

**Figure 2.**
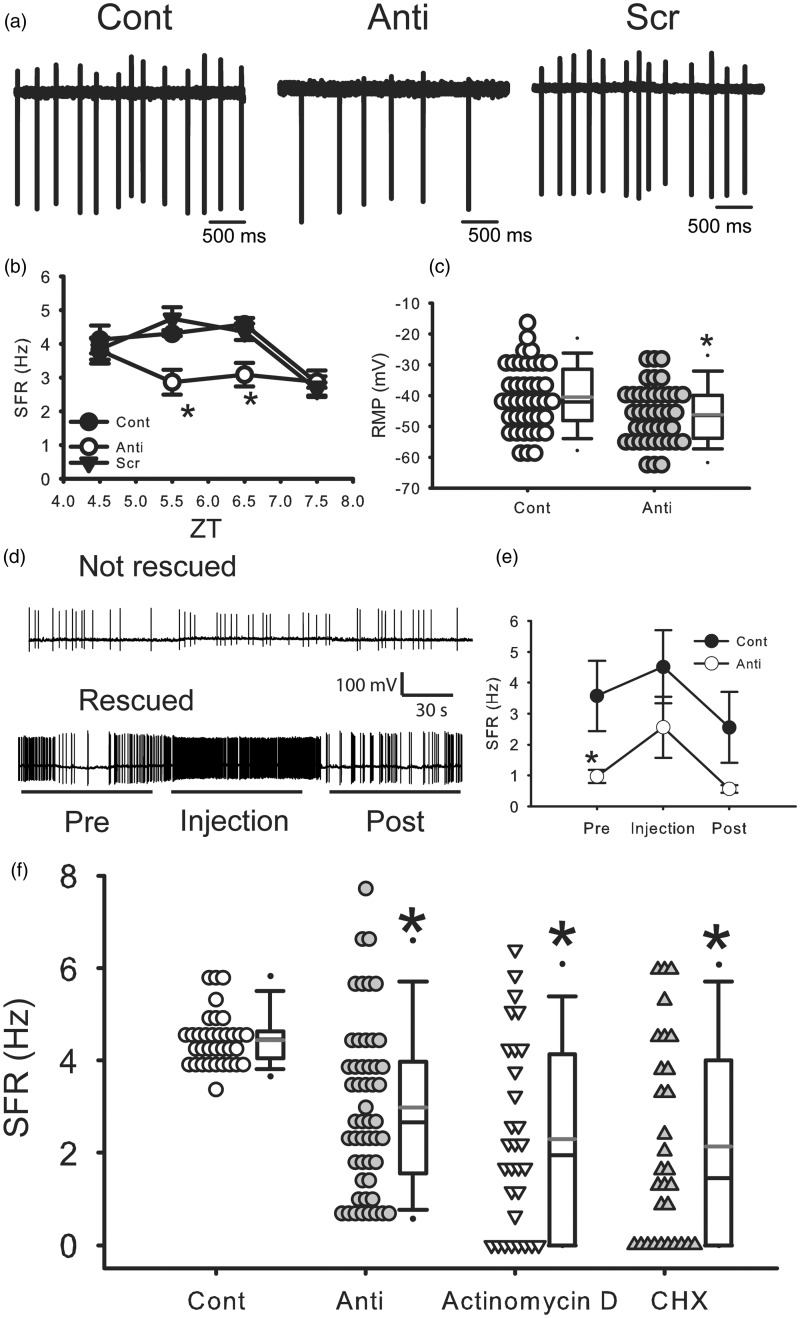
Antisense ODN for *Per1* reduced firing rate in dorsal SCN neurons during the day. (a) Representative examples illustrating the reduced firing rates by the antisense ODN for *Per1* (10 µM). Cont: control, Anti: antisense ODN for *Per1*, and Scr: scrambled ODN. (b) Average firing rate for each group ( ± *SE*). *Significant difference (*p* < .05) analyzed by two-way ANOVA followed by Holm-Sidak method for multiple comparisons (vs. Cont). For each group *n* = 17–28 cells from 4 mice at each time point (in total, 271 cells from 48 mice). (c) Average resting membrane potential for each group ( ± *SE*). *Significant difference (*p* < .05) analyzed by unpaired Student’s *t*-test. For each group, *n* = 40 cells from 30 to 37 mice. (d) Representative examples illustrating the effects of current injections on SFR under antisense ODN for *Per1*. (e) Average firing rate for each group (±*SE*). *Significant difference (*p* < .05) analyzed by two-way repeated measures ANOVA followed by Holm-Sidak method for multiple comparisons (vs. Cont). For each group, *n* = 10 cells from 7 to 9 mice. (f) Effects of actinomycin D and cycloheximide (CHX) on SFR in the SCN during daytime. Actinomycin D solution (0.8 µM final concentration), CHX (final 36 µM), or water (as a control) was added to the culture medium 3 h before the start of recordings. The SFR was measured by loose-patch method. Average firing rate for each group (±*SE*). *Significant difference (*p* < .05) analyzed by one-way ANOVA followed by Dunn’s method for multiple comparisons (vs. Cont). For each group, *n* = 30–51 cells from 4 to 8 mice. Cont and Anti data are the same as (b).

To examine the impact of the transient reduction of PER1 on the electrical activity in the SCN, we sampled SFR in dSCN neurons at different time points (2–6 h) after the end of the ODN treatment (ZT 2–4; Supplemental Figure 1, electrophysiology 1). Two-way ANOVA revealed significant differences of main effects (time: *F*(3, 259) = 10.101, *p* < .01, treatment: *F*(2, 259) = 7.975, *p* < .01). Also there was significant interaction between Time × Treatment (*F*(6, 259) = 2.842, *p* < .05). The antisense ODN treatment caused a significant reduction in SFR at ZT 5.5 and 6.5 (Figure 2(a) and (b), two-way ANOVA followed by Holm-Sidak method for multiple comparisons, vs. Cont, ZT 5.5: *p* < .05, ZT 6.5: *p* < .05). The SFR returned to control level by 3.5 h after the treatment ended (ZT 7.5). In PER1 protein levels, time course of PER1 reduction showed lowest level at ZT 5, but PER1 level returned to control level at ZT 6. On the other hand, SFR was lower at ZT 5.5–6.5. Scrambled ODN did not alter the SFR at any time (Figure 2(a) and (b); two-way ANOVA followed by Holm-Sidak method, n.s.). This analysis assumes that every neuron is independent as is typically for electrophysiological recordings. We also examined the impact of the antisense treatment by averaging all of the values for the individual neurons to come up with an average SFR per mouse. Here too, we found that the antisense ODN significantly reduced SFR (Cont: 4.4 ± 0.2 Hz, *n* = 8 mice, anti: 3.1 ± 0.4, *n* = 8 mice, unpaired Student’s *t*-test, *p* < .05). We examined resting membrane potential of dSCN neurons during ZT 5 to 7 and found that the membrane potential was significantly hyperpolarized in *Per1* antisense-treated group (Figure 2(c), unpaired Student’s *t*-test, *p* < .05, Cont: 40 cells from 30 mice, −40.5 ± 1.7 mV, anti: 40 cells from 37 mice, −46.2 ± 1.5 mV).

Next, we sought to determine if direct current injection into SCN neurons could overcome the decreased SFR observed under antisense treatment. Using the current-clamp recording technique in the whole cell patch configuration, we performed current injections in dSCN neurons. For these experiments, SFRs were measured in current clamp mode for 1 min at first. Next, 1 min square depolarizing current (5 pA) was injected to examine the effects of depolarization on SFR. When the membrane resistance is 1 GΩ, this current injection causes a 5 mV depolarization. After current injection, SFRs were recorded without current injection. The results were mixed. Most cells (7 cells, 70%) exhibited a dramatic increase in firing (>140%) by the current injection (5 pA), but some cells (3 cells, 30%) did not respond (Figure 2(d) and (e)). This suggests that a specific ionic mechanism may be altered by the antisense ODN for *Per1*. These results demonstrate that most antisense-treated neurons still had the capacity to fire action potentials at the normal frequency.

If the suppression of transcription and translation of *Per1* is critical for SFR regulation, broad transcriptional and translational inhibitors should produce the same effects. Therefore, we examined the effects of broad transcriptional (Actinomycin D) and translational (cycloheximide) inhibitors using the same protocol (Supplemental Figure 1). Both Actinomycin D and cycloheximide significantly reduced the SFR in the SCN during day (Figure 2(f), Kruskal-Wallis ANOVA on ranks followed by Dunn’s method, *H*_3_ = 31.272, Actinomycin D: *p* < .05 vs. Cont, cycloheximide: *p* < .05 vs. Cont). These results indicate that global transcriptional and translational inhibitors mimicked the effects of antisense ODN for *Per1* on SFR.

To determine whether the circadian phase was shifted by antisense ODN, we examined the effects of antisense on the rhythm in PER2::LUC in SCN explants. We found that the reduction in PER1 did not result in significant phase shifts measured in the next day (Cont: projected ZT (pZT) 10.5 ± 1.2, *n* = 8, Anti: pZT13.5 ± 1.1, *n* = 8, unpaired Student’s *t*-test, n.s.).

### Antisense ODN Against *Per1* Reduced [Ca^2+^]i Levels in Cell in the SCN During Daytime

**Figure 3.**
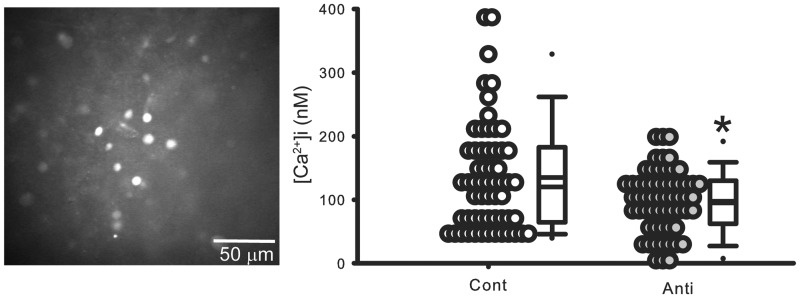
Antisense ODN for *Per1* reduced [Ca^2+^]i levels in cells in the SCN during daytime. In these experiments, resting [Ca^2+^]i levels were estimated in SCN neurons in brain slices with antisense ODN for *Per1* and compared with data obtained from the control group. Animals were sacrificed at ZT 0. Each cell is sampled only once. Left: Representative pictures in Fura2 imaging in the SCN. Right: Dot density plot showing that [Ca^2+^]i levels in SCN cells were reduced by antisense ODN for *Per1*. Cont: control, Anti: antisense ODN for *Per1*. *Significant difference (*p* < .05) analyzed by unpaired Student’s *t*-test. Bottom: Histograms illustrating the distribution of [Ca^2+^]I levels. For each group (±*SE*), *n* = 59 cells from four mice.

In SCN neurons, changes in electrical activity are directly reflected in changes in [Ca^2+^]i levels (Irwin and Allen, 2013). Using [Ca^2+^]i imaging techniques and the same protocol as described earlier, we evaluated the impact of reducing PER1 on resting [Ca^2+^]i levels in SCN neurons (protocol shown in Supplemental Figure 1). We found [Ca^2+^]i levels in the SCN neurons treated with the antisense ODN were significantly reduced (Mann-Whitney *U* test, *p* < .05) compared with Cont (Figure 3). We estimated the resting [Ca^2+^]i levels to be 135 ± 11 nM in Cont and 98 ± 6 nM in the treated neurons. Thus, we confirmed that reducing PER1 leads to lower [Ca^2+^]i levels in SCN neurons.

### Antisense ODN Against *Per1* Reduced BK Currents in the SCN during Daytime

**Figure 4.**
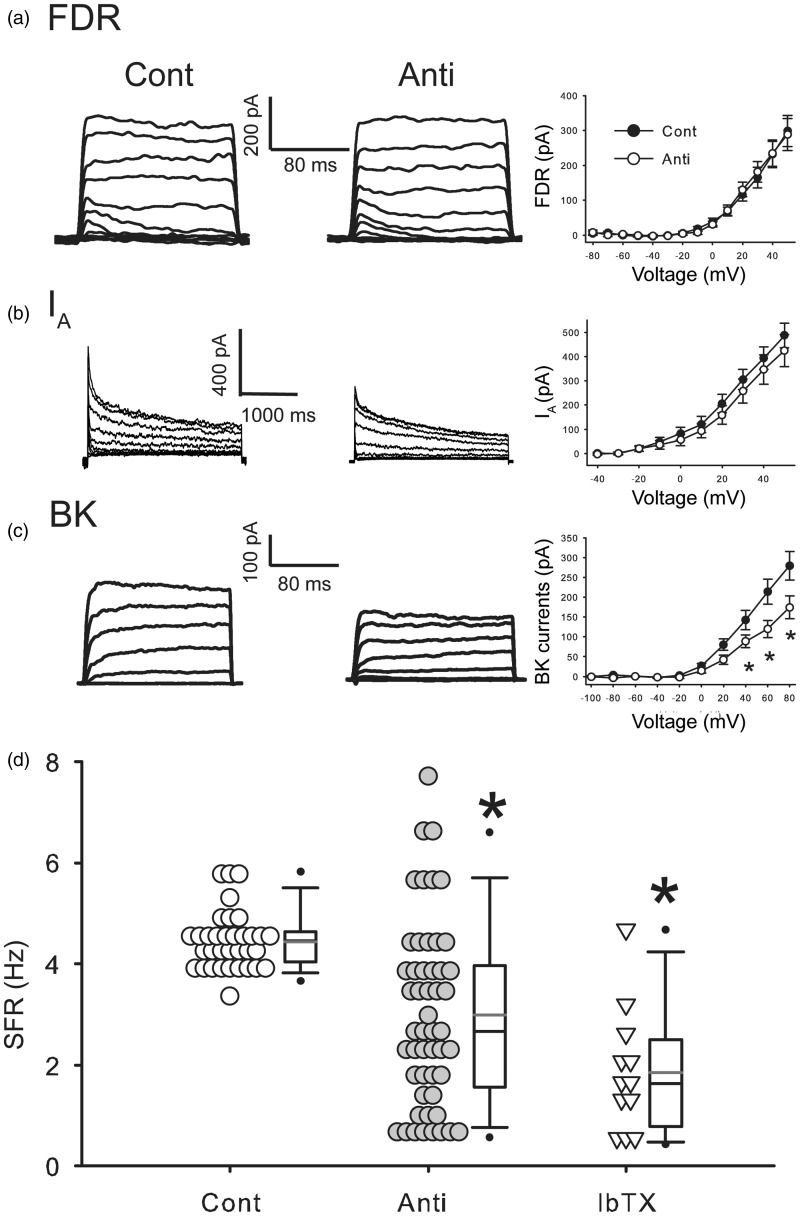
Antisense ODN for *Per1* reduced BK currents in the SCN during daytime. (a) Left, representative examples showing that application of antisense ODN for *Per1* (10 µM) did not decrease the magnitude of fast delayed rectifier (FDR) currents in the SCN neurons. Right, current-voltage (I–V) relationship of FDR currents in SCN neurons. Cont: control, Anti: antisense ODN for *Per1*. For each group (±*SE*), *n* = 20 cells from 10 mice. (b) Left, representative examples showing the I_A_ currents. Right, I–V curve of I_A_ measured in SCN neurons. For each group (±*SE*), *n* = 11–15 cells from 4 to 13 mice. (c) Left, representative examples showing BK currents. Right, I–V curve of BK current measured in SCN neurons. *Significant difference (*p* < .05) analyzed by two-way repeated measures ANOVA followed by Holm-Sidak method for multiple comparisons (vs. Cont). For each group (±*SE*), *n* = 20–21 cells from 15 to 18 mice. (d) Average firing rate of control, antisense ODN for *Per1*, and iberiotoxin (IbTX). *Significant difference (*p* < .05) analyzed by one-way ANOVA followed by Dunn’s method for multiple comparisons (vs. Cont). For each group *n* = 12–51 cells from 6 to 8 mice. Cont and Anti data are the same as Figure 2(b). The currents were normalized by capacitance.

A set of voltage-gated potassium currents controls the SFR of SCN neurons (Colwell, 2011) and we sought to identify the specific potassium currents that are altered by the reduction of PER1. Whole cell patch clamp recordings were used to measure FDR, I_A_, and BK currents in the SCN during the day (ZT 5–7; protocol shown in Supplemental Figure 1). Each of these three currents has been suggested to have a role in the modulation of SFR in SCN neurons during the daytime. Antisense ODN against *Per1* did not alter the magnitude of the FDR (Figure 4(a), two-way repeated measures ANOVA, main effects; treatment: n.s.) or I_A_ currents (Figure 4(b), FDR: two-way repeated measures ANOVA, main effects; treatment: n.s.). Importantly, the treatment did significantly reduce the magnitude of the BK current (Figure 4(c)). Two-way repeated measures ANOVA revealed main effects of an interaction of Treatment × Voltage (*F*(9, 441) = 3.112, *p* < .01). Antisense ODN against *Per1* significantly reduced the magnitude of BK currents at 60 mV (post hoc: Holm-Sidak, *p* < .05, vs. Cont), 80 mV (Holm-Sidak, *p* < .05, vs. Cont). Finally, we examined if BK currents blocker IbTX effects on the SFR in the SCN. The application of the BK blocker IbTX reduced daytime SFR to a similar degree as the antisense ODN (Figure 4(d), one-way ANOVA followed by Dunn’s method, *p* < .05, vs. Cont). There was no significant differences in the variance of SFR recorded between antisense and IbTX-treated groups (equal variance test, *F* = 2.19, *p* = .08). These results indicate that the antisense ODN for *Per1* rapidly and selectively reduces the BK current in the SCN.

## Discussion

*Per1* is one of the core clock genes expressed in high levels in the mouse SCN (Sun et al., 1997; Tei et al., 1997). The *Per1* mRNA levels exhibit a robust circadian rhythm with transcript levels starting to rise near dawn and peak expression in the middle of day (CT 6–8). The protein is delayed a few hours with PER1 protein peaking around CT 10 in the mouse SCN (LeSauter et al., 2003). In the present study, we demonstrate that antisense ODN application during the rising phase of protein levels (ZT 2–4) was able to transiently reduce PER1 levels for 2 h (Figure 1). The treatment was selective and did not alter levels of the closely related PER2 (Figure 1). The transient knock down of PER1 was not sufficient to alter the phase of the molecular clockwork as measured by PER2::LUC bioluminescence. This enabled us to examine, for the first time, the impact on the membrane of transiently reducing PER1 in SCN neurons. The finding is important as it shows that the transient reduction of this clock gene rapidly impacts membrane events. It fits with the prior observation that the *Per1* KO mice show arrhythmic or low amplitude rhythms in clock gene expression (Pendergast et al., 2009). Both of these results stand in contrast to the *in vivo* analysis of the *Per1* mutants that show normal rhythms in wheel running behavior and SCN neural activity rhythms (Pendergast et al., 2009; Takasu et al., 2013). The difference in effect between *in vitro* and *in vivo* analysis is becoming a common theme in circadian rhythms research where the SCN circadian clock has been shown to be quite resilient and has both genetic (DeBruyne et al., 2007) and circuit level (Liu et al., 2007) mechanisms to protect against mutations. Part of the appeal of the rapid antisense approach is that it circumvents the possibility of genetic compensation.

We found that antisense ODN treatment caused a transient reduction (2–3 h) in SFR (Figure 2(a) and (b)). When PER1 expression (Figure 1(a) and (b)) and SFR (Figure 2(f)) are compared, the variability of the SFR is relatively large. In our work, we are measuring PER1 expression in the whole SCN but measuring SFR from single neurons. Prior work has shown that there is a correlation between *Per1* levels and electrical activity in single SCN neurons. In *Per1*::GFP mice, the temporal dynamics of transcriptional activation of *Per1* can be optically followed by monitoring green fluorescent protein (GFP) fluorescence. In these mice, there is a direct correlation between the frequency of action potentials and the level of *Per1* expression in SCN neurons (Kuhlman et al., 2003; Quintero et al., 2003). It is not clear whether *Per1* expression is correlated to the SFR per se or more directly a reflection of the underlying membrane depolarization as another study using the same mice reported a high GFP signal in SCN neurons that were so depolarized as to be unable to generate action potentials (Belle et al., 2009).

Our hypothesis is that circadian rhythms in electrical activity are driven, in part, through rhythmic changes in *Per1* expression. This hypothesis is supported by our observation that transient decreases in PER1 levels result in a reduction in SFR. However, the relationship between *Per1* expression and membrane potential may be complex in that studies at a population level reveal that membrane hyperpolarization, by lowering the extracellular concentration of K^+^ in SCN cultures, reversibly abolishes the rhythmic expression of *Per1* (Lundkvist et al., 2005). These studies, along with work in *Drosophila* (Nitabach et al., 2002; Sheeba et al., 2008), suggest that electrical activity of pacemaker neurons impacts the molecular clockwork, and that keeping SCN cells in an appropriate voltage range may be required for the generation of circadian rhythmicity of clock gene expression at the single-cell level. Thus, the relationship between *Per1*expression and membrane potential may be bidirectional.

Our findings raise new questions about the underlying mechanism by which reducing PER1 could alter membrane activity within hours. While changes in trafficking or posttranslational modifications of ion channels seem to offer a likely mechanistic explanation, we found that broad spectrum inhibitors of both transcription and translation mimicked the impact of the antisense ODN treatment (Figure 2(f)). Most of the SCN neurons still had the capacity to generate action potentials at the normal firing rate as direct current injection increased SFR in some but not all of the neurons (7 out of 10 SCN neurons tested; Figure 2(d) and (e)). Next we went on to examine [Ca^2+^]i levels within SCN neurons. SCN neurons show a clear [Ca^2+^]i rhythm (Colwell, 2000; Enoki et al., 2012; Brancaccio et al., 2013) which, like the electrical activity, is high during daytime and low during nighttime. The antisense ODN treatment caused a reduction in resting [Ca^2+^]i during the middle of the day (Figure 3). Presently, we do not know whether the reduction in [Ca^2+^]i occurs prior to the impact on electrical activity or whether the decrease in electrical activity drives the reduced [Ca^2+^]i. There is evidence that the core clock protein Cryptochrome can alter the cAMP signaling cascade through an interaction with G-proteins (Zhang et al., 2010), so it is possible that PER1 could have a similar role in the [Ca^2+^]i regulatory process.

A set of voltage-gated potassium currents control the SFR of SCN neurons (Colwell, 2011). We sought to identify the specific K^+^ currents that are altered by the reduction of PER1 using whole-cell patch clamp recordings. The magnitudes of both the FDR (Itri et al., 2005; Kudo et al., 2011) and I_A_ (Itri et al., 2010; Granados-Fuentes et al., 2012) currents vary with the circadian cycle and are important regulators of SCN SFR during the day. However, our data demonstrate that neither of these currents is altered by the reduction in PER1 (Figure 4). In contrast, the BK currents were significantly reduced. Prior work has shown that the BK current is important for SFR in SCN neurons (Pitts et al., 2006; Kent and Meredith, 2008; Montgomery and Meredith, 2012) and expression of rhythmic behavior (Meredith et al., 2006). The gene that encodes the pore-forming subunit of the BK channel (*Kcnma1*) and the BK channel are rhythmically expressed in the SCN (Panda et al., 2002; Meredith et al., 2006; Pitts et al., 2006). This body of work suggests that the BK current is important for both the nocturnal hyperpolarization in SCN neurons but also for the regulation of daytime firing rate (see Montgomery and Meredith, 2012). We confirmed that blocking the BK channel with IbTX mimics the effect of the antisense (Figure 4) as has been previously reported (Pitts et al., 2006). Therefore, our results support a model in which PER1 regulates expression of the BK current, either directly or indirectly, through a mechanism dependent on transcription and translation of proteins.

The molecular clockwork that generates rhythms in PER1 is widely expressed in the body including electrically active cells in the islets, heart, and brain. There is a lack of a mechanistic understanding of how this molecular feedback loop interacts with the membrane to produce circadian rhythms in electrical activity that is important for these cell populations. Clearly, the signals traveling to and from this molecular feedback loop must travel through the membrane, but it is not known how the molecular feedback loop drives the rhythm in electrical membrane processes. In the present study, we provide evidence that the molecular clock regulates firing rate and the magnitude of the BK current in SCN neurons. Certainly, other currents are also impacted by the molecular clockwork, but here we provide a concrete example that the reduction of PER1 rapidly alters a critical current in the SCN. Our data provide new insights into the underlying mechanisms by which the molecular rhythmicity ultimately impact mammalian physiology and behavior. Ultimately, much of the work of the central nervous system is affected through the generation and transmission of electrical signals. Thus, it is critical to understand the relationship between intracellular molecular feedback loops giving rise to circadian rhythms and the transmission of rhythmic electrical information throughout the nervous system. Consequenctly, there are compelling reasons to better understand the mechanisms underpinning the interaction between the molecular clock and electrical activity.

## Summary

In this study, we demonstrate for the first time that transiently and selectively reducing the levels of the clock gene *Period1* (*Per1*) decreases electrical activity in the mouse SCN.

## Supplementary Material

Supplementary material
