# Supplementary figures and images for: The Circadian Clock Gene Period1 Connects the Molecular Clock to Neural Activity in the Suprachiasmatic Nucleus
Source: ASN Neuro. 2015 Oct 30;7(6):1759091415610761. doi: 10.1177/1759091415610761 (PMC4710129; doi:10.1177/1759091415610761)

Supplemental figure 1

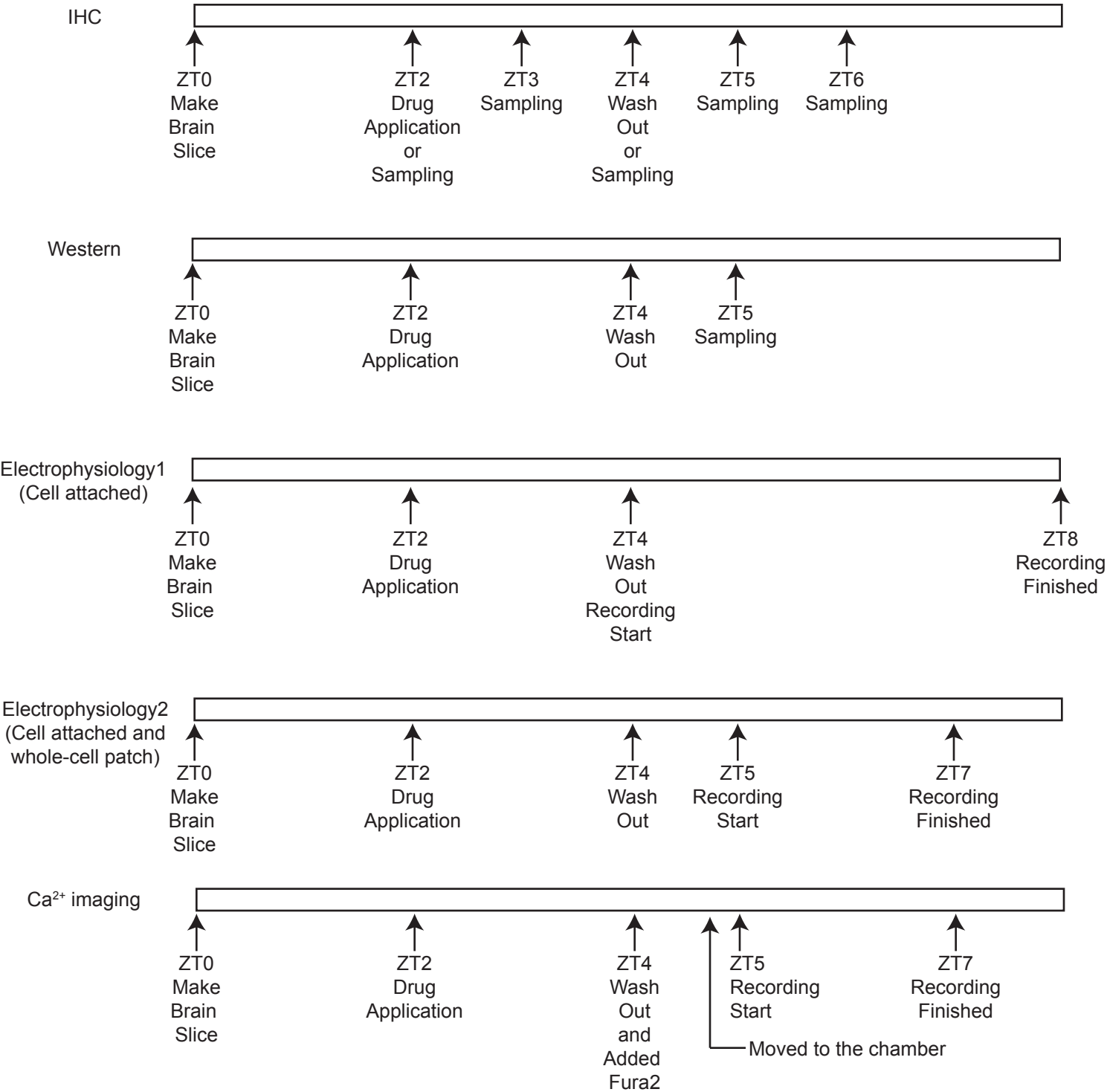

Supplement: Supplementary material [file Supplemental_Figure_1_761.pdf]
